# Susceptible gene of stasis-stagnation constitution from genome-wide association study related to cardiovascular disturbance and possible regulated traditional Chinese medicine

**DOI:** 10.1186/s12906-015-0761-x

**Published:** 2015-07-14

**Authors:** Kuo-Chin Huang, Hung-Jin Huang, Ching-Chu Chen, Chwen-Tzuei Chang, Tzu-Yuan Wang, Rong-Hsing Chen, Yu-Chian Chen, Fuu-Jen Tsai

**Affiliations:** Graduate Institute of Chinese Medicine, China Medical University, Taichung, 40402 Taiwan; Department of Integration of Traditional Chinese and Western Medicine, China Medical University Hospital, Taichung, 40447 Taiwan; Department of Chinese Pharmaceutical Sciences and Chinese Medicine Resources, College of Pharmacy, China Medical University, Taichung, 40402 Taiwan; School of Chinese Medicine, College of Chinese Medicine, China Medical University, Taichung, 40402 Taiwan; Division of Endocrinology and Metabolism, Department of Medicine, China Medical University Hospital, Taichung, 40447 Taiwan; Human Genetic Center, Department of Medical Research, China Medical University Hospital, 40402 Taichung, Taiwan; Research Center for Chinese Medicine & Acupuncture, China Medical University, Taichung, 40402 Taiwan; Department of Biotechnology, Asia University, Taichung, 41354 Taiwan; Department of Medical Genetics, Medical Research and Pediatrics, China Medical University Hospital, No. 2, Yuh Der Road, Taichung, Taiwan

**Keywords:** Type 2 diabetes, Genome-wide association study, Body constitution, Traditional Chinese medicine, Type 2 diabetes, Molecular dynamics (MD) simulation

## Abstract

**Background:**

This study identified susceptible loci related to the Yu-Zhi (YZ) constitution, which indicates stasis-stagnation, found in a genome-wide association study (GWAS) in patients with type 2 diabetes and possible regulated traditional Chinese medicine (TCM) using docking and molecular dynamics (MD) simulation.

**Methods:**

Non-aboriginal Taiwanese with type 2 diabetes were recruited. Components of the YZ constitution were assessed by a self-reported questionnaire. Genome-wide SNP genotypes were obtained using the Illumina HumanHap550 platform. The world’s largest TCM database (http://tcm.cmu.edu.tw/) was employed to investigate potential compounds for PON2 interactions.

**Results:**

The study involved 1,021 unrelated individuals with type 2 diabetes. Genotyping data were obtained from 947 of the 1,021 participants. The GWAS identified 22 susceptible single nucleotide polymorphisms on 13 regions of 11 chromosomes for the YZ constitution. Genotypic distribution showed that *PON2* on chromosome 7 was most significantly associated with the risk of the YZ constitution. Docking and MD simulation indicated 13-hydroxy-(9E_11E)-octadecadienoic acid was the most stable TCM ligand.

**Conclusions:**

Risk loci occurred in *PON2*, which has antioxidant properties that might protect against atherosclerosis and hyperglycemia, showing it is a susceptible gene for the YZ constitution and possible regulation by 13-hydroxy-(9E_11E)-octadecadienoic acid.

**Electronic supplementary material:**

The online version of this article (doi:10.1186/s12906-015-0761-x) contains supplementary material, which is available to authorized users.

## Background

Differences exist between traditional Chinese medicine (TCM) and conventional western medicine. These differences include not only the treatment approach (such as herbal medicine and acupuncture in TCM) but also the underlying theories. A principle component of TCM theory is the concept of constitution, which provides a method for classifying patients according to type. Constitution demonstrates individual differences in structure and function, temperament, and environmental adaptability. Patients with different constitutions have different susceptibilities, development, and prognoses for certain diseases. According to *Huang Di Nei Jing*, a textbook of TCM internal medicine written approximately 2,000 years ago, a certain constitution is partially developed from congenital factors [1]. This view is similar to “personalized medicine,” which highlights the genetic background for disease susceptibility. Genetic studies to investigate the congenital factors of constitution are increasing in the post-genome era. Chen et al. reported that allele frequencies of human leukocyte antigens including DPB1*0501 in the Yin-deficiency group (Frequency 51.6 % vs 35.6 %, relative risk 1.9), DRB1*09012 in the Phlegmwetness group (Frequency 23.4 % vs 12.8 %, relative risk 2.1), and DQB1*03032 in the Qi-deficiency (Frequency 22.2 % vs 8.1 %, relative risk 3.2) and Phlegm-wetness groups (Frequency 19.9 % vs 8.1 %, relative risk 2.8) differ significantly from those in the normal constitution [2]. Wang et al. conducted an expression array and identified 785 upregulated genes and 954 downregulated genes in the Yang-deficiency constitution, compared with those in normal individuals. The most significant enriched Gene Ontology Cluster of upregulated genes is “response to stress” which contained interleukin factors and their receptors. The most significant enriched Gene Ontology Cluster of downregulated genes is “nucleobase, nucleoside, nucleotide and nucleic acid metabolism” which contained thyroid hormone receptor signal pathway [3]. A study of polymorphisms further identified the biased distribution of single nucleotide polymorphisms (SNPs) in *PPARD* (peroxisome proliferator-activated receptors delta) rs2267669 and rs2076167 and *APM1* (adipose most abundant gene transcript 1) rs7627128 and rs1063539 in the Yang-deficiency constitution; *PPARD* rs2076167 and APM1 rs266729 and rs7627128 in Phlegm-wetness constitution; and in *PPARG* (peroxisome proliferator-activated receptors gamma) Pro12Ala in the Yin-deficiency constitution [4]. Gene expression is influenced by environmental factors in the posttranscriptional process and candidate gene studies are limited to a certain viewing region.

Cardiovascular disease is a major complication in patients with diabetes mellitus (DM), especially in those with type 2 diabetes, resulting in both comorbidity and mortality [5]. One meta-analysis reported that DM tends to double the risk of cardiovascular disease [6]. Screening for high cardiovascular risk in patients and providing more effective protection for these patients are important in clinical practice. The Yu-Zhi (YZ) constitution in TCM indicates stasis and stagnation, which expressed dull, lusterless skin color; dry, cracked, scaly or tough skin; dull purple lips or tongue; localized pain or numbness; knotted, intermittent, or uneven pulse. It is one of the body constitutions that tend to express blood stasis syndrome (BSS), a morbid state caused by blood circulation disturbance, included extravasated blood, blood circulating sluggishly, or blood congested in viscera, that may turn into pathogenic factors. BSS is usually considered a link to cardiovascular complications. A study of hospitalized patients with coronary artery disease (CAD) noted that BSS was the most common TCM pattern in over three-quarters of the patients [7]. The risk of BSS increases with the carotid intima-media thickness in patients with dyslipidemia [8]. According to TCM theory, BSS constitutes the main mechanism of cardiovascular diseases, including diabetic cardiovascular complications. The pathogenesis of BSS includes microcirculation disturbance, abnormal hemorheological factors, and hemodynamic changes. Some small samples molecular studies also detected differences in cell surface antigens and gene expression between those with BSS and healthy controls [9, 10]. As mentioned earlier, congenital factors are considered to be a principal component of constitutional formation; possible genetic variations underlying the YZ constitution are of interest. In the present study, a genome-wide association study was conducted to identify susceptible loci related to the YZ constitution in patients with type 2 diabetes. Genes related to the susceptible loci are considered susceptible genes of the YZ constitution. Computer-aided drug design (CADD) has been widely used in studies investigating new treatments [11–13], and could help accelerate the development of leading drugs [14, 15]. CADD could be employed to approaches in the design of drugs for anti-inflammation [16], anti-virus [17, 18], pain regulation [19], weight loss [20, 21], stroke therapy [22–24], and cancer therapy [25–28]. Hence, we employed a TCM database (http://tcm.cmu.edu.tw) [29] and natural compounds to conduct virtual screening for proteins of susceptible genes by molecular docking to find potential TCM or natural compounds. Then we performed molecular dynamics (MD) simulation to study the protein-ligand interactions and stabilized conformations for the top candidates.

## Methods

### Study participants

Our study population comprised adult patients with type 2 diabetes in Taiwan. Patients willing to participate in a genetic study in nonaboriginal Taiwanese were recruited from the outpatient clinic of China Medical University Hospital (Taiwan) between September 2006 and June 2007. Type 2 diabetes was diagnosed according to the criteria of the 1997 American Diabetes Association [30]. Patients with type 1 diabetes, gestational diabetes, or maturity-onset diabetes of the young were all excluded. This research was approved by the China Medical University Hospital Institutional Review Board, and all participants gave informed consent.

### Data collection

Patient information (age, sex, age at diagnosis of diabetes, smoking history) was collected by a questionnaire. Systolic and diastolic blood pressures were obtained by averaging two measurements with a resting interval of at least 5 min. Patients with hypertension were defined as having a systolic pressure of more than 130 mmHg, and a diastolic pressure of more than 80 mmHg, or those who were receiving antihypertensive agents. The body height and weight of subjects (wearing light clothing and no shoes) were measured by experienced research staff. The body mass index was calculated by dividing the body weight (kg) by the square of the body height (m). Blood samples were drawn between 8:00 and 10:00 a.m. after the patients had fasted overnight, and separated serum was stored at −70 °C until assayed. Fasting plasma glucose was detected by the hexokinase method. Serum total cholesterol, triglycerides, high- (HDL) and low-density lipoprotein (LDL) cholesterol, creatinine, and uric acid levels were measured by standard laboratory methods. High-sensitivity C-reactive protein was measured by immunoturbidimetry (Integra 700; Roche, Mannheim, Germany). Hemoglobin A1c was gauged by the high-performance liquid chromatography method (HLC-723G7; TOSOH Bioscience, Tokyo, Japan). The albumin-to-creatinine ratio (ACR) was obtained from a morning spot urine test and data were categorized as normoalbuminuria (ACR ≦ 30 mg/g), microalbuminuria (30 mg/g < ACR < 300 mg/g) or macroalbuminuria (ACR ≧ 300 mg/g). Biochemical analyses were performed at the Taipei Institution of Pathology.

### Yu-Zhi constitution questionnaire

The YZ body constitution was assessed by a questionnaire, which was developed using a psychometrically sound method as reported previously [31], and comprises eight self-reported symptomatic items(Supp.) [32]. Each item was assessed on a 5-point Likert scale (never, occasionally, sometimes, often, and always). The YZ score was obtained by a summation of the scores of the eight items, and a higher score indicated stronger intensity of the YZ constitution.

### Genotyping

Genomic DNA was extracted from peripheral blood mononuclear cells for a genome-wide association study. The procedures for genomic DNA extraction, whole genome genotyping, genotype calling and quality control have been described previously [33]. PUREGENE DNA isolation kit (Gentra Systems, Minneapolis, MN) was use to extract genomic DNA from peripheral blood mononuclear cells, and Illumina HumanHap550-Duo BeadChips was used to perform the whole genome genotyping in deCODE genetics (Reykjavı’k, Iceland). The standard procedure implemented in BeadStudio, with default parameters suggested by the platform manufacturer was used to perform Genotype calling. Genotyping validation was performed using the Sequenom iPLEX assay (Sequenom MassARRAY system; Sequenom, San Diego, CA, USA). By examining several summary statistics, quality control of the genotype data was performed. First, by calculating the ratio of loci having heterozygous calls on the X chromosome, sex of the patients was double-checked. Second, total successful call rate and the minor allele frequency were also calculated for each SNP. The exclusion situations for SNPs were as follows: no polymorphism, a total call rate of < 95 %, or a minor allele frequency of < 5 % and a total call rate < 99 %. A total of 560,184 SNPs were genotyped, 38,700 SNPs were excluded due to quality control criteria, 12,723 SNPs were excluded due to Hardy-Weinberg equilibrium principle (P <0.0001) and 508,761 SNPs were used in final analysis.

### Statistical analysis

Data from continuous variables were expressed as mean ± standard deviation and categorical variables were expressed as percentages. Participants were divided into high and low YZ score groups according to the median score (YZ score = 10). Differences between the high and low YZ score groups were compared using Student’s *t*-test for continuous variables and the *χ*^2^ test for categorical variables. Association analysis was carried out to compare allele frequency and genotype distribution between the high and low YZ score groups using 6 single point methods for each SNP: genotype, allele, trend (Cochran-Armitage test), additive, dominant, and recessive models using PLINK (PLINK 1.07, http://pngu.mgh.harvard.edu/~purcell/plink/contact.shtml#cite) and SAS (SAS Institute Inc., 100 SAS Campus Drive, Cary, NC 27513–2414, USA).

The associated SNPs were selected from those at least showing *p*-values < 10^−5^ under the most significant test statistic obtained from any of the 6 statistical models. We then used a multivariate logistic regression model to determine the genotype odds ratios (ORs) and 95 % confidence intervals (CIs) of associated SNPs in the best model. For ORs, *p*-values < 0.05 were considered statistically significant.

### Structure preparation and docking study

Because the PON2 structure is not available in the PDB database, the sequence of PON2 (UniProt ID: Q15165) was obtained from the UniProt database for 3D structure modeling. The sequence of PON2 was submitted to the I-TASSER server [34–36] (iterative threading assembly refinement algorithm) to generate a 3D structure of PON2. For 3D structure validation, we also employed a Ramachandran plot [37], profile-3D (Discovery Studio Client v2.5; Accelrys, San Diego, CA, USA.) and PONDR-FIT [38] (DisProt, http://www.disprot.org/) to validate the PON2 modelling structure. The residue His114 of PON2 was regarded as the binding site for screening TCM compounds based on protein-ligand interaction [39]. We used the LigandFit module of DS 2.5 to calculate docking poses of TCM compounds in the PON2 protein structure. Each binding pose of the TCM ligands was generated by Monte-Carlo techniques. The minimization of TCM compounds utilized the CHARMm force field [40]. The minimization step in each docking pose was performed with 1000 steps of the Steepest Descent and Conjugate Gradient. The generated conformations of ligands were docked into the defined binding site of the PON2 modeling structure. All of the TCM compounds with docking poses had various scoring functions including the piecewise linear potential (−PLP1, −PLP2), potential of mean force (−PMF), and Dock Scores.

### Molecular dynamics simulation

The MD simulation was carried out by the GROMACS 4.5.5 package [41] to simulate the dynamic condition of the protein-ligand complex from the docking results of PON2. The edge of the box between the protein complexes was set as 1.2 nm. We choice the charmm27 force field for the simulation system [42]. The protein-ligand complex containing water molecules was placed by TIP3P model cubic cells. Non-bonded interactions included Coulomb terms and van der Waals (VDW). The particle mesh Ewald method [43, 44] was used to define Coulomb interactions as electrostatic in this experiment, and the cut-off distance of VDW residues was set at 1.4 nm. The linear constraint solver algorithm was used to fix all bond lengths among all atoms of the protein-ligand complexes. Topology files and parameters of TCM compounds in PON2 complexes were generated from the SwissParam web server [45] for the GROMACS simulation. For ion setting of solvent concentrations, the Na^+^ and Cl^−^ ions were randomly replaced the water molecules in the solvent system with a concentration of 0.145 M. The minimization of energy was used to stabilize the protein-ligand complex by the steepest descent method with 5000 steps, followed by equilibration performed under position restraints with 1 ns for balance of the water molecules between PON2 complexes. The condition of equilibration was under constant temperature dynamics (NVT type) at a temperature of 310 K. In the final step, a production run was performed for 20,000 ps with constant pressure and temperature dynamics (NPT type). The temperature of the production run was set at 310 K. All frames of MD conformation were sampled every 20 ps for trajectory analysis under GROMACS4.5.5.

## Results

This study enrolled 1,0*2*1 patients with type 2 diabetes who were 20 years old or older. Participants were divided into a high YZ score group and low YZ score group according to the median (numbers of high YZ score: low YZ score = 583: 438). The patient information and clinical characteristics of the high and low YZ score groups are summarized in Table 1. Compared with the low YZ score group, the high YZ score group was significantly older (61.3 ± 11.4 vs 59.7 ± 10.5 years; *p* = 0.020), included more women (53.5 vs 47.3 %; *p* = 0.048), had a longer duration of diabetes (11.9 ± 7.4 vs 10.8 ± 6.8 years; *p* = 0.017), and displayed poorer control of serum glucose (hemoglobin A1c 8.0 ± 1.5 vs 7.8 ± 1.4 %; *p* = 0.015).

**Table 1:** Characteristic and clinical profiles of the study subjects

	High YZ score (*n* = 583)	Low YZ score (*n* = 438)	*p-*value
Mean age (years)	61.3 ± 11.4	59.7 ± 10.5	0.020*
Male (%)	46.5	52.7	0.048*
Diabetes duration (years)	11.9 ± 7.4	10.8 ± 6.8	0.017*
Hypertension ratio (%)	73.9	79	0.060
Current smoking (%)	18.3	18.0	0.917
Body mass index (kg/m2)	25.6 ± 4.0	25.7 ± 3.5	0.722
Fasting plasma glucose (mmol/L)	8.0 ± 2.5	7.9 ± 2.3	0.590
Hemoglobin A1c %	8.0 ± 1.5	7.8 ± 1.4	0.015*
Total cholesterol (mmol/L)	4.9 ± 1.1	4.9 ± 1.0	0.845
Triglycerides (mmol/L)	1.9 ± 1.6	1.8 ± 1.3	0.322
HDL cholesterol (mmol/L)	1.2 ± 0.4	1.3 ± 0.4	0.181
LDL cholesterol (mmol/L)	3.1 ± 1.0	3.1 ± 0.9	0.912
hs-CRP (mg/L)	3.1 ± 6.5	3.0 ± 10.4	0.899
Creatinine (μmol/L)	80.4 ± 61.9	79.2 ± 47.4	0.747
Uric acid (μmol/L)	374.9 ± 113.1	374.9 ± 101.2	0.888
Normoalbuminuria (%)	56.7	61.6	0.101
Microalbuminuria (%)	28.7	28.0	
Macroalbuminuria (%)	14.6	10.3	

YZ, Yu-Zhi; HDL, High-density lipoprotein; LDL, low-density lipoprotein; hs-CRP, high-sensitivity C-reactive protein; ACR, urine albumin-to-creatinine ratio

Values are mean ± SD, or percentages

*p* value: *t*-test or *χ*2 test to sex, current smoking, hypertension ratio and ACR

* *p* < 0.05

**Fig. 1:**
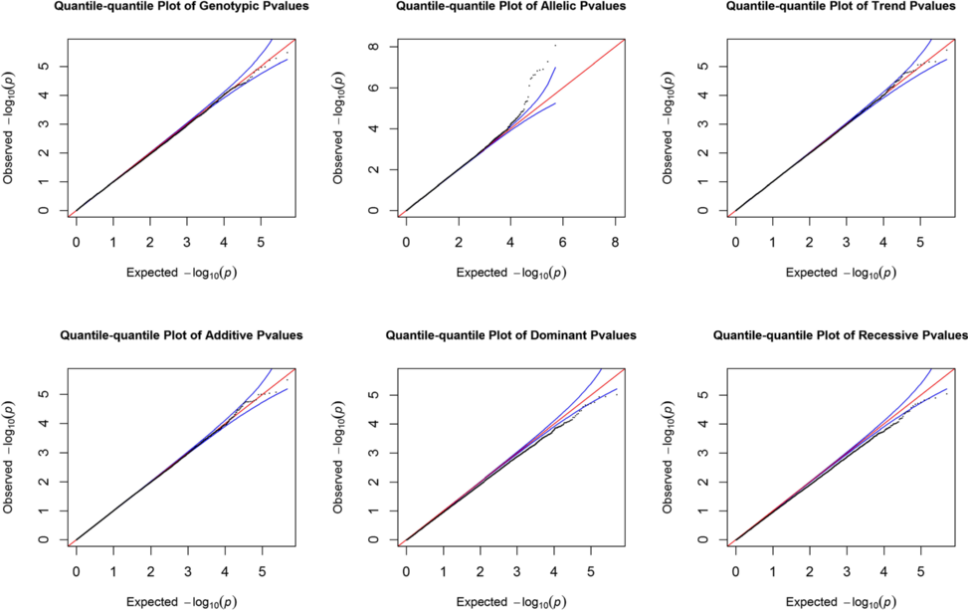
Quantile–quantile plots of genotype, allele, trend (Cochran-Armitage test), additive, dominant, and recessive models. Observed *p*-values were compared with the expected *p*-values under the null distribution for each model

**Fig. 2:**
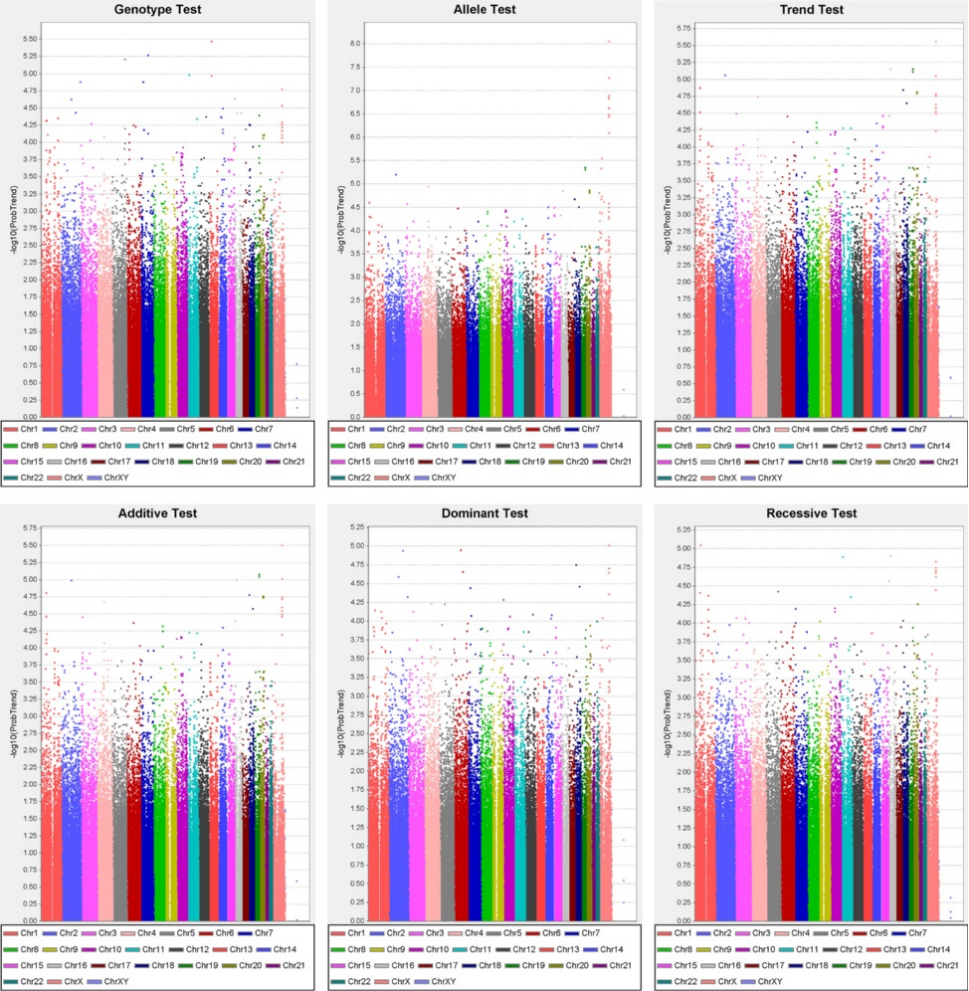
Manhattan plots of genotype, allele, trend (Cochran-Armitage test), additive, dominant, and recessive models

Genotyping data were obtained from 947 (numbers of high YZ score: low YZ score = 539: 408) of the 1,021 participants using Illumina HumanHap550duov3 chips. Quantile–quantile plots for each model were shown that the distribution of observed *p*-values deviated from expected *p*-values in Fig. 1. Manhattan plots of *p*-values across all chromosomes for each model were shown in Fig. 2. SNPs in autosomal chromosomes with a *p*-value < 9.8 × 10^−8^ were not detected in all the 6 statistical models. Table 2 summarizes the SNPs selected from results showing *p*-values < 10^−5^ under the most significant test statistic obtained from any of the 6 statistical models. However, the false discovery rate was high. The SNP rs7694118 is located on chromosome 4 in the 5’ untranslated region (UTR) of *PCDH10* (protocadherin 10). The SNP rs7493 is located on chromosome 7 in an exon region of *PON2* (paraoxonase 2) and is in complete linkage disequlibrium with rs2299263 and rs17166875 (D’ = 1.0, r^2^ = 1.0) in intron regions. The SNP rs4526895 is in tight linkage disequlibrium with rs12865228 (D’ = 1; r^2^ = 0.97). Two of the SNPs are located in an intron of *FREM2* (*FRAS1* [Fraser syndrome 1] related extracellular matrix protein 2) on chromosome 13. The SNP rs8093481was strongly associated with the YZ constitution (*p* = 9.64 × 10^−7^) and in complete linkage disequilibrium with rs11660953 (D’ = 1; r^2^ = 1). Two of the SNPs are located in an intron region of the *PIEZO2* (piezo-type mechanosensitive ion channel component 2) gene on chromosome 18. The SNP rs4801958 is located on chromosome 19 in an exon region of the *ZNF665* (zinc finger protein 665) and is completely linked with rs12460170 (D’ = 1.0, r^2^ = 1.0), which is also in an exon region. It is also tightly linked with rs12971799 (D’ = 1.0, r^2^ = 0.989) and rs1133146 (D’ = 1.0, r^2^ = 0.989) in the 3’ UTR, and with rs4803055 (D’ = 0.987, r^2^ = 0.824) in an intron region of the ZNF665.

**Table 2:** Summary of the SNPs associated with high Yu-Zhi score in type 2 diabetes

					RA frequency						
dbSNP ID	Chr.	Position (Mb)	Gene	RA* (NRA)	High YZ score	Low YZ score	*p*-value^‡^ (Best model)	-log (*p*-value)	best model	Effect size (95 % CI)	FDR
rs12036718	1p	48.3	Unknow	T(C)	0.174	0.103	8.10 × 10^−6^	5.09	Trend	1.68 (0.95 -2.99)	1.00
rs1932064	1p	70.8	Unknow	T(C)	0.788	0.700	6.18 × 10^−6^	5.21	Dominant	1.62 (1.05 -2.51)	0.97
rs8179355	1p	70.9	Unknow	C(A)	0.782	0.699	7.53 × 10^−6^	5.12	Recessive	1.54 (1.25 -1.89)	0.86
rs9633289	1p	70.9	Unknow	T(A)	0.217	0.298	6.18 × 10^−6^	5.21	Recessive	1.55 (1.11 -2.15)	0.95
rs7565310	2q	128.9	Unknow	A(G)	0.484	0.380	8.63 × 10^−6^	5.06	Trend	1.53 (1.27 -1.84)	0.83
rs7694118	4q	134.3	PCDH10	C(T)	0.698	0.663	6.31 × 10^−6^	5.20	Genotype	1.30 (0.85 -1.99)	1.00
rs164368	5q	160.6	Unknow	T(C)	0.803	0.785	6.14 × 10^−6^	5.21	Genotype	1.12 (0.89 -1.40)	1.00
rs7493	7q	94.9	PON2	C(G)	0.180	0.177	5.33 × 10^−6^	5.27	Genotype	1.06 (0.74 -1.51)	1.00
rs2299263	7q	94.9	PON2	A(G)	0.180	0.177	5.33 × 10^−6^	5.27	Genotype	1.06 (0.74 -1.51)	1.00
rs17166875	7q	94.9	PON2	T(C)	0.180	0.177	5.33 × 10^−6^	5.27	Genotype	1.02 (0.81 -1.29)	1.00
rs12865228	13q	38.2	FREM2	G(T)	0.429	0.414	3.38 × 10^−6^	5.47	Genotype	1.06 (0.88 -1.28)	1.00
rs4526895	13q	38.2	FREM2	C(T)	0.428	0.414	1.79 × 10^−6^	5.75	Genotype	1.38 (0.93 -2.04)	1.00
rs17118382	14q	82.8	Unknow	A(G)	0.815	0.730	9.75 × 10^−6^	5.01	Allele	1.45 (1.03 -2.04)	0.98
rs194045	16p	29.2	Unknow	G(A)	0.943	0.888	6.31 × 10^−6^	5.20	Trend	2.13 (1.51 -3.02)	0.83
rs8093481	18p	10.7	PIEZO2	A(G)	0.757	0.656	9.64 × 10^−7^	6.02	Trend	1.79 (1.16 -2.77)	0.93
rs11660953	18p	10.7	PIEZO2	T(C)	0.756	0.656	1.60 × 10^−6^	5.79	Trend	1.79 (1.16 -2.77)	0.93
rs1133146	19q	58.4	ZNF665	A(G)	0.990	0.956	4.77 × 10^−6^	5.32	Allele	1.52 (1.01 -2.29)	0.99
rs12971799	19q	58.4	ZNF665	C(T)	0.700	0.599	4.77 × 10^−6^	5.32	Allele	1.49 (0.99 -2.25)	1.00
rs4801958	19q	58.4	ZNF665	T(C)	0.700	0.599	4.77 × 10^−6^	5.32	Allele	1.56 (1.29 -1.89)	0.83
rs12460170	19q	58.4	ZNF665	G(A)	0.699	0.600	7.56 × 10^−6^	5.12	Allele	1.56 (1.29 -1.89)	0.83
rs4803055	19q	58.4	ZNF665	C(T)	0.737	0.639	4.87 × 10^−6^	5.31	Allele	1.56 (1.02 -2.38)	0.98
rs871913	20p	16.1	Unknow	A(G)	0.099	0.045	8.24 × 10^−6^	5.08	Trend	1.86 (0.78 -4.44)	1.00

Table 3 shows the results of multiple logistic regression analysis of the genotypic distribution of susceptible SNPs in patients with high YZ scores among the high and low YZ score groups. The results showed that 20 SNPs in 11 regions of 10 chromosomes were significantly associated with high YZ scores in the best model, after controlling for age, sex, diabetes duration, and hemoglobin A1c. The risk genotypes were defined by homozygous risk alleles (higher allele frequency in the high YZ score group than in the low YZ score group). Under the best model, the risky CC genotype of rs7493, AA genotype of rs2299263 and TT genotype of rs17166875 within the *PON2* gene in chromosome 7 were associated with a high YZ score, with an 8.62-fold (95 % CI, 2.60-28.52) increase in risk. The risky AA genotype of rs8093481 and TT genotype of rs11660953 within the *PIEZO2* gene in chromosome 18 increased the risk of a high YZ score 2.33-fold (95 % CI, 1.31-4.14). The risky GG genotype of rs194045 in chromosome 16 increased the risk of a high YZ score 2.2-fold (95 % CI, 1.52-3.19). The risky AA genotype of rs7565310 in chromosome 2 was associated with a 2.11-fold (95 % CI, 1.49-2.99) increase in risk.

**Table 3:** Genotypic distribution between high and low Yu-Zhi score, and adjusted odds ratios of SNPs associated with high Yu-Zhi Score in type 2 diabetes

	Gene	dbSNP ID	Risk allele	Genotype^#^	High YZ score (%)	Low YZ score (%)	OR (95 % CI) ^‡^
Chr.	Unknown	rs12036718	T	CC	70.3	78.9	1.00(Ref)
1p				TT + CT	29.7	21.1	1.68 (0.87-3.24)
	Unknown	rs1932064	T	CC + CT	43.2	57.9	1.00(Ref)
1p				TT	56.8	42.1	1.78 (1.01-3.13)*
	Unknown	rs8179355	C	AA + AC	38.0	52.6	1.00(Ref)
1p				CC	62.0	47.4	1.78 (1.36-2.31)*
	Unknown	rs9633289	T	AA + AT	40.7	54.9	1.00(Ref)
1p				TT	59.3	45.1	1.73 (1.13-2.63)*
	Unknown	rs7565310	A	GG + AG	75.1	86.5	1.00(Ref)
2q				AA	24.9	13.5	2.11 (1.49-2.99)*
	PCDH10	rs7694118	C	TT	4.1	13.3	1.00(Ref)
4q				CC + CT	95.9	86.7	0.27 (0.10-0.72) *
	Unknown	rs164368	T	CC	6.7	2.2	1.00(Ref)
5q				TT + CT	93.3	97.8	0.30 (0.14-0.64)*
	PON2	rs7493	C	GG + CG	94.2	99.3	1.00(Ref)
7q				CC	5.8	0.7	8.62 (2.60-28.52)*
	PON2	rs2299263	A	GG + AG	94.2	99.3	1.00(Ref)
7q				AA	5.8	0.7	8.62 (2.60-28.52)*
	PON2	rs17166875	T	CC + CT	94.2	99.3	1.00(Ref)
7q				TT	5.8	0.7	8.62 (2.60-28.52)*
	FREM2	rs12865228	G	TT	29.2	38.7	1.00(Ref)
13q				GG + GT	70.8	61.3	1.59 (1.21-2.10)*
	FREM2	rs4526895	C	TT	29.2	38.7	1.00(Ref)
13q				CC + CT	70.8	61.3	1.59 (1.21-2.10)*
	Unknown	rs17118382	A	GG	4.0	8.8	1.00(Ref)
14q				AA + AG	96.0	91.2	0.42 (0.19-0.95)*
	Unknown	rs194045	G	AA + AG	10.6	21.3	1.00(Ref)
16p				GG	89.4	78.7	2.20 (1.52-3.19)*
	PIEZO2	rs8093481	A	GG + AG	39.6	58.9	1.00(Ref)
18p				AA	60.4	41.1	2.33 (1.31-4.12)*
	PIEZO2	rs11660953	T	CC + CT	39.6	58.9	1.00(Ref)
18p				TT	60.4	41.1	2.33 (1.31-4.12)*
	ZNF665	rs1133146	A	GG + AG	48.6	64.2	1.00(Ref)
19q				AA	51.4	35.8	1.94 (1.10-3.42)*
	ZNF665	rs12971799	C	TT + CT	48.6	64.2	1.00(Ref)
19q				CC	51.4	35.8	1.94 (1.10-3.42)*
	ZNF665	rs4801958	T	CC + CT	50.1	63.2	1.00(Ref)
19q				TT	49.9	36.8	1.67 (1.28-2.18)*
	ZNF665	rs12460170	G	AA + AG	50.2	63.0	1.00(Ref)
19q				GG	49.8	37.0	1.65 (1.26-2.15)*
	ZNF665	rs4803055	C	TT + CT	41.4	60.6	1.00(Ref)
19q				CC	58.6	39.4	2.25 (1.27-3.97)*
	Unknown	rs871913	A	GG	84.5	91.6	1.00(Ref)
20p				AA + AG	15.5	8.4	2.18 (0.88-5.40)

Case number: High Yu-Zhi score = 538, Low Yu-Zhi score = 409

Chr, chromosome; dbSNP ID, SNP database indentification; YZ, Yu-Zhi; CI, confidence intervals of odds ratio; PCDH10, protocadherin 10; PON2, paraoxonase 2; FREM2, FRAS1 related extracellular matrix protein 2; PIEZO2, piezo-type mechanosensitive ion channel component 2; ZNF665, zinc finger protein 665

^#^Better of dominant or recessive model

^‡^Adjusted by age, sex, DM duration and hemoglobin A1c

*Significant difference of genotype distribution between high YZ group and low YZ group (*p* < 0.05)

The *ZNF665* gene’s completely linked SNPs, the risky AA genotype of rs1133146 and the GG genotype of rs12971799, were associated with a 1.94-fold (95 % CI, 1.10-3.42) increase in risk. The risky TT genotype of rs4801958 and GG genotype of rs12460170 within the *ZNF665* gene in chromosome 19 were associated with a 1.67-fold (95 % CI, 1.28-2.18) and 1.65-fold (95 % CI, 1.26-2.15) increase in risk for a high YZ score, respectively. The risky CC genotype of rs4803055 was associated with a 2.25-fold increase in risk (95 % CI, 1.27-3.97).

The risky TT genotype of rs1932064 in chromosome 1 was associated with a 1.78-fold increase in risk (95 % CI, 1.01-3.13). Two tightly linked SNPs, the CC genotype of rs8179355 and the TT genotype of rs9633289, carried a 1.78-fold (95 % CI, 1.36-2.31) and 1.73-fold (95 % CI, 1.13-2.63) increase in risk, respectively. The SNP rs12865228 within the *FREM2* gene on chromosome 13 and its completely linked SNP rs4526895 were associated with a 1.59-fold (95 % CI, 1.21-2.10) increase in risk.

By contrast, rs7694118 in *PCDH10* of chromosome 4, rs164368 in chromosome 5 and rs17118382 of chromosome 14 decreased the risk of high YZ score by 73 % (OR 0.27; 95 % CI, 0.10-0.72), 70 % (OR 0.30; 95 % CI, 0.14-0.64), and 58 % (OR 0.42; 95 % CI, 0.19-0.95), respectively, in the dominant model, after controlling for age, sex, diabetes duration, and hemoglobin A1c. For the remaining SNPs, no differences emerged between patients with high versus low YZ scores.

**Fig. 3:**
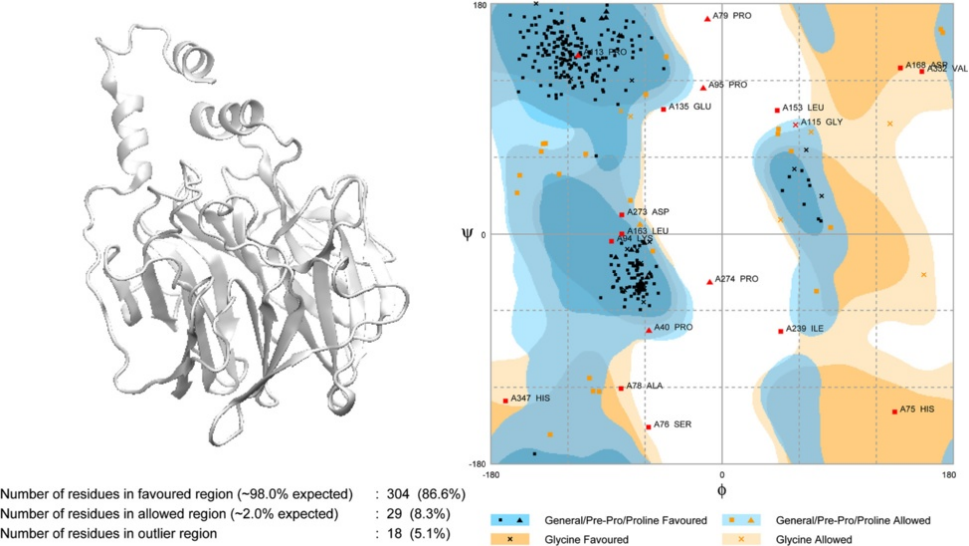
Ramachandran plot of the PON2 modelling structure. The modelling structure was built from the ITASSER server. There are 86.6 % and 5.1 % of residues of PON2 in the *favoured and disfavoured regions, respectively

**Fig. 4:**
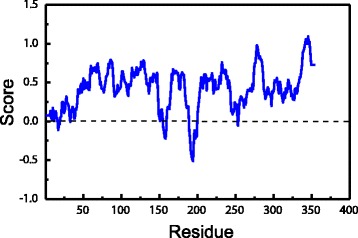
3D-profile of the best PON2 modelling structure. A score above zero indicates the modelling residue is reliable

**Fig. 5:**
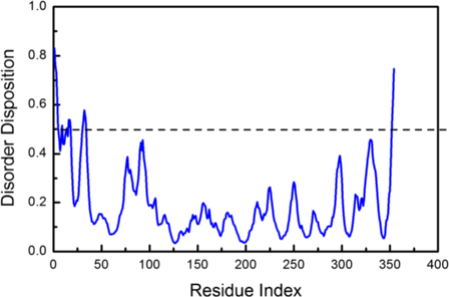
The disorder analysis of the PON2 sequence by PONDR-FIT prediction. Values of disorder disposition under 0.5 denote ordered residues

The PON2 protein was built from the I-TASSER server and we validated the simulated PON2 structural residues by Ramachandran plot (Fig. 3), with 86.6 % of residues of PON2 located in the favoured region, and only 5.1 % of residues in the disfavoured regions. We also used 3D-profiling to observe the reliability of each residue. Most PON2 residues had validation scores with positive score values (Fig. 4). The score for the active residue, His144, displayed that it was reliable from the modelling structure, indicating that this key residue was not affected by the docking screen process. For protein disorder analysis (Fig. 5), most of the sequence (including residue His114) revealed that the disorder disposition was below 0.5. The prediction of disorder analysis illustrated that the PON2 protein is a folded structure, and may not affect TCM compounds during the docking process [46, 47].

**Fig. 6:**
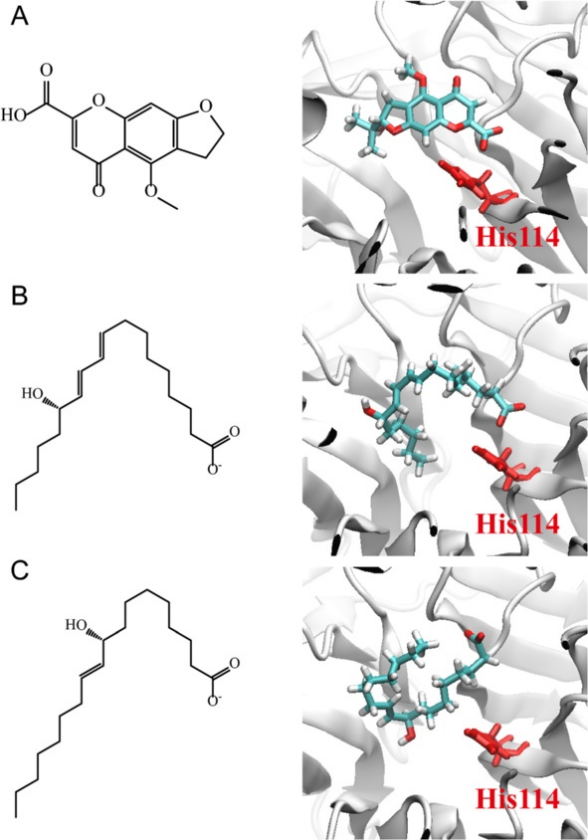
The docking poses of the top three candidates from TCM database screening: (**a**) divaricatacid, (**b**)13-hydroxy-(9E_11E)-octadecadienoic acid, and (**c**) 9-hydroxy-(10E)-octadecenoic acid. The active residue His114 is in red

The docking analysis was based on -PLP1, −PLP2, −PMF, and Dock Scores to select the docking poses of TCM compounds from database screening. From the scoring analysis, we analysed the top ten TCM ligands with high -PMF scores as candidates (Table 4). Furthermore, we found the docking poses of the top three candidates, divaricatacid, 13-hydroxy-(9E_11E)-octadecadienoic acid, and 9-hydroxy-(10E)-octadecenoic acid, were very close to the key residue His114 (Fig. 6). The docking poses showing that the three candidates can interact with His114, and have the potential to activate PON2 for antioxidation. In a further study, we performed MD simulation to observe the stability of TCM candidates in the PON2 structures under dynamic condition.

**Table 4:** Scoring functions of the top ten candidates from TCM database screening of PON2 protein structure

Name	-PLP1	-PLP2	-PMF	Dock Score
Divaricatacid	66.24	64.27	139.09	59.236
13-hydroxy-(9E_11E)-octadecadienoic acid	76.84	79.89	137.40	27.758
9-hydroxy-(10E)-octadecenoic acid	68.05	70.50	132.62	34.441
10-hydroxy-(8E)-octadecenoic acid	73.47	76.87	132.32	39.635
11-hydroxy-(9Z)-octadecenoic acid	86.85	85.40	130.44	32.403
Divaricataester A	75.03	66.69	124.91	55.187
8-hydroxy-(9E)-octadecenoic acid	51.03	59.33	124.80	36.94
Benzyl-O-beta-D-glucopyranoside	57.08	53.02	122.42	47.813
Alismorientols A	52.95	58.02	118.54	26.339
1-O-beta-D-glucopyranosyloxy-3-methylbut-2-en-1-ol	55.51	47.42	117.38	41.243
PMF; potential of mean force; PLP, piecewise linear potential

**Fig. 7:**
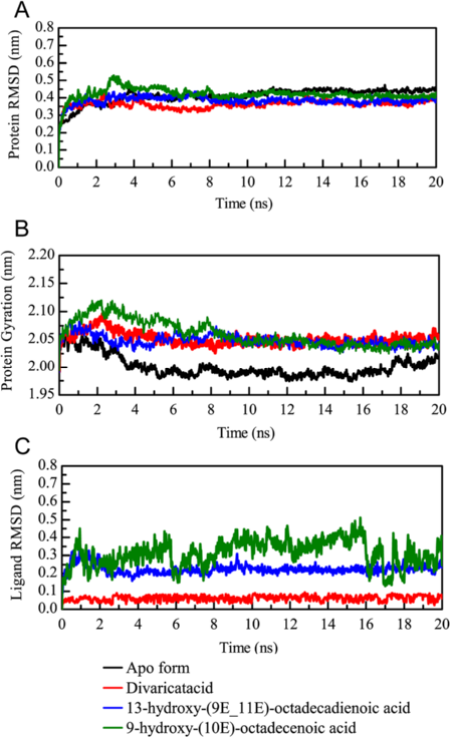
Plots of (**a**) protein root mean square deviation (RMSD) and (**b**) radius of the gyration and ligand RMSD values in the analysis of PON2 systems during a simulation time of 20 ns

**Fig. 8:**
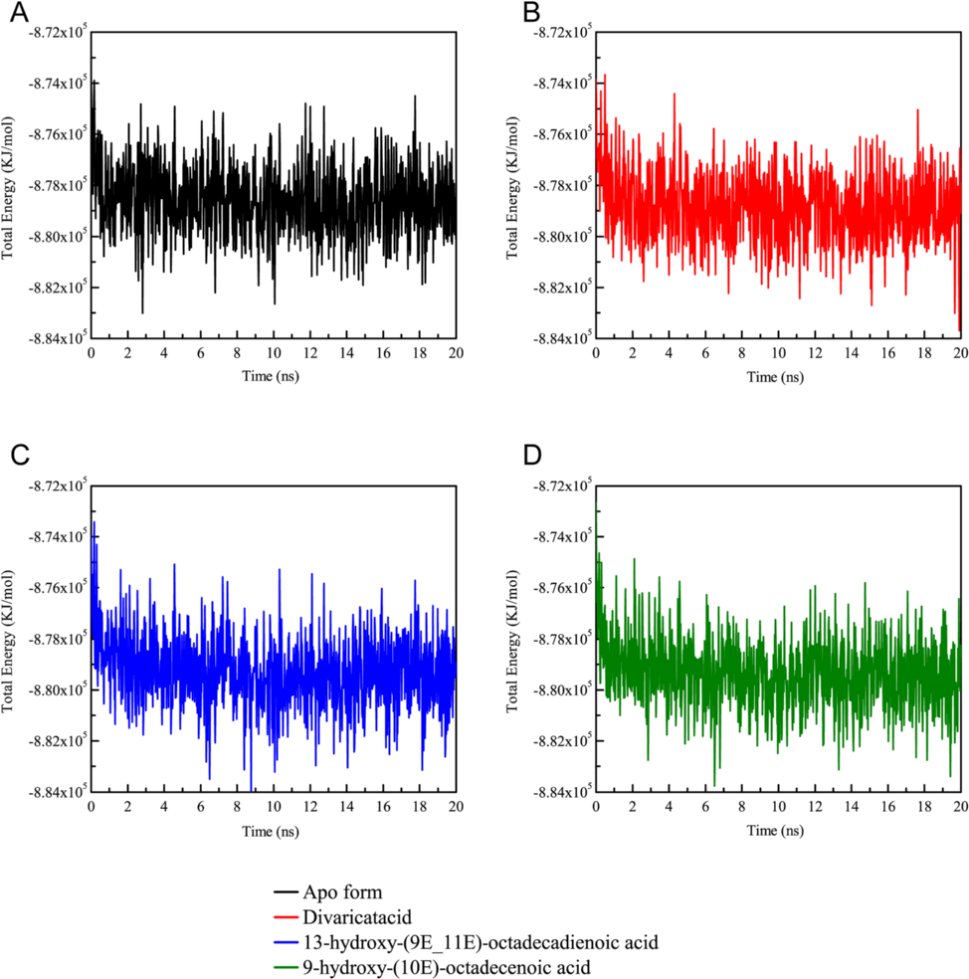
Total energy of the PON2 systems for a simulation time of 20 ns

For the trajectory analysis of the MD simulation, the root mean square deviation (RMSD) and gyration of protein atoms were used to observe the stability of the protein structure for PON2 and protein-ligand complexes. The value of the protein RMSD was around 0.2 and 0.3 nm from 2 ns to 20 ns (Fig. 7a). The apo form of the PON2 and PON2 complexes with TCM candidates revealed stable fluctuation during all of the MD simulation time, and 20 ns of simulation time facilitated all simulation systems into constant conditions. The gyration of the PON2 structure was state in 2.05 from 8 ns to 20 ns (Fig. 7b). The apo form revealed a decreasing value of gyration, which was more compact than the PON2 complexes with TCM candidates, illustrating that TCM compounds not leave aware from the PON2 structure during all MD simulation times. For the ligand RMSD, we can found that 9-hydroxy-(10E)-octadecenoic acid displayed fluctuating RMSD values from 6 ns to 20 ns. Divaricatacid and 13-hydroxy-(9E_11E)-octadecadienoic acid revealed stable ligand RMSDs during the overall simulation time (Fig. 7c). Hence, 9-hydroxy-(10E)-octadecenoic acid may not be suitable to interact with the PON2 structure. The total energy of the PON2 complexes (Fig. 8) with divaricatacid, 13-hydroxy-(9E_11E)-octadecadienoic acid, and 9-hydroxy-(10E)-octadecenoic acid was −8.74 × 10^5^ during the initial simulation time (from 0 ns to 2 ns), but in the final step of the simulation time, the total energy tended to be stable at −8.78 × 10^5^ which was similar to the apo form of PON2. The results for total energy show that all systems of PON2 are stable after 20 ns of MD simulation time.

**Fig. 9:**
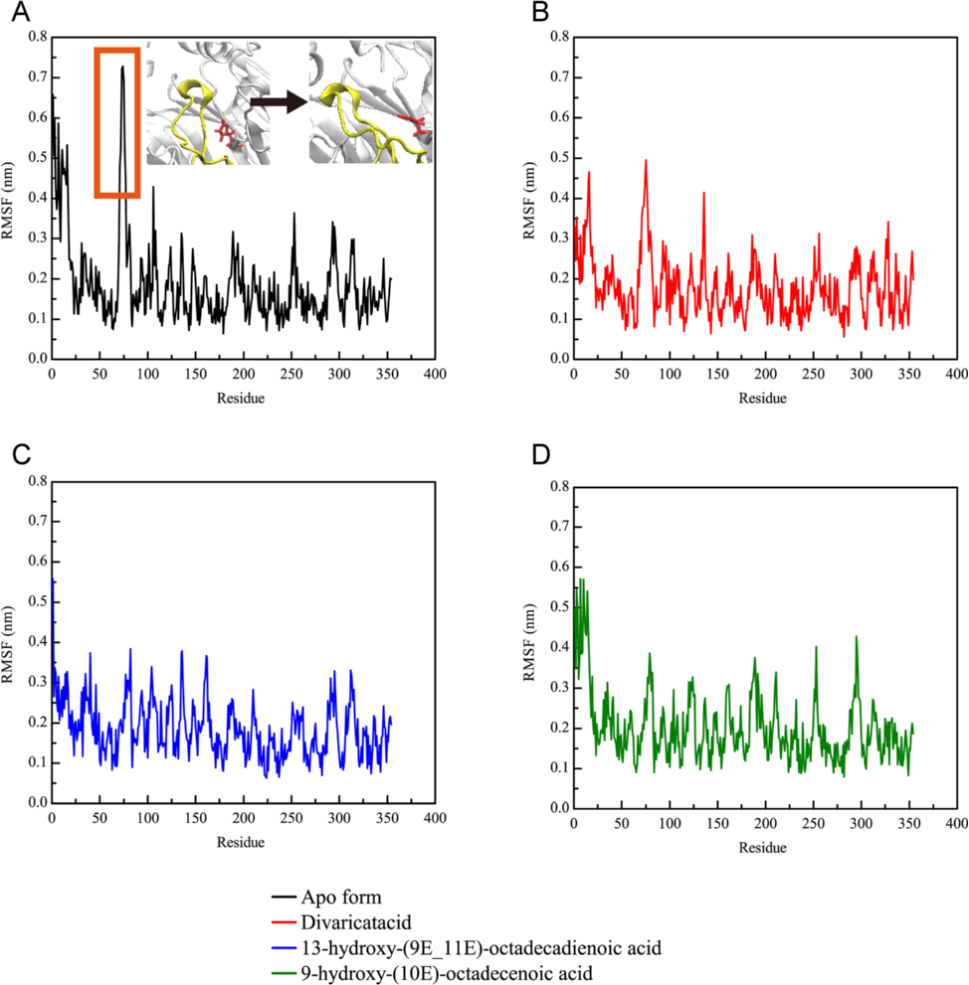
Root mean square fluctuation values for each residue of the PON2 structure in the apo form and protein-ligand complexes for a simulation time of 20 ns

**Fig. 10:**
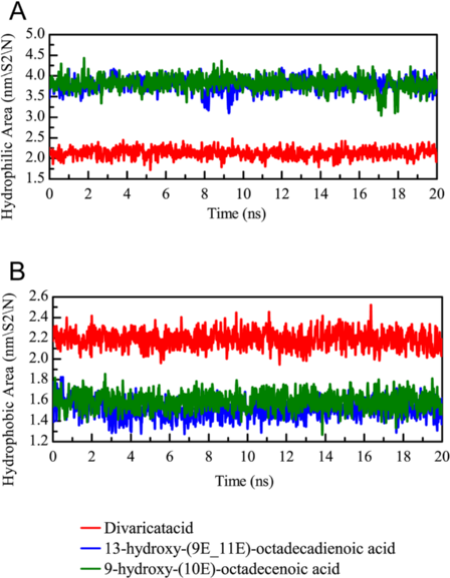
Solvent accessible hydrophilic (**a**) and hydrophobic (**b**) surface areas for the PON2 structure with TCM compounds

We further calculated the root mean square fluctuation (RMSF) values for each residue of the PON2 protein structure (Fig. 9). Interestingly, we found the residues revealed high fluctuations from 50 to 100 on the apo form of PON2 (Fig. 9a). The high RMSF values indicate the residue was flexible at all simulation times. The PON2 structure with docked TCM compounds was more stable than the apo form, which suggests that TCM compounds can stabilize the protein structure in the protein-ligand complex type. Because of the flexible residues on the apo form of PON2 near the key residue His114, reducing the variation of these residues denotes that TCM compounds could tightly interact with the PON2 structure. We also calculated the solvent accessible hydrophobic and hydrophilic surface areas for the three TCM compounds. The results show that divaricatacid is suitable for hydrophobic solvents, because the hydrophilic areas of 13-hydroxy-(9E_11E)-octadecadienoic acid and 9-hydroxy-(10E)-octadecenoic acid were wider than that of divaricatacid (Fig. 10).

**Fig. 11:**
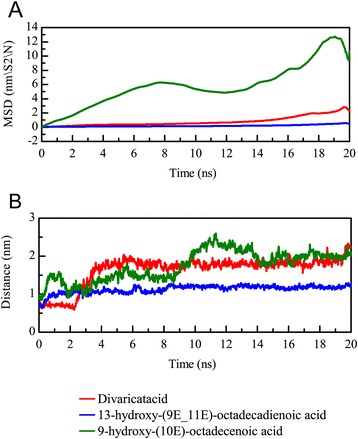
The mean square displacement (MSD) of different ligands (**a**) for a simulation time of 20 ns. A high MSD value indicates the ligand has migrated farther from the initial site. The distance between the centers of mass of RbAp48 and each ligand (**b**) during a simulation time of 20 ns

To analyze the migration of each TCM compound during the MD simulation time, we computed the mean square displacement (MSD) value to measure the variation of each ligand in the PON2 structure. 9-hydroxy-(10E)-octadecenoic acid displayed a significantly higher MSD value during the 20 ns simulation time than the other two candidates (Fig. 11a). In addition, we further calculated the distance between PON2 and the three candidates. The distance between 13-hydroxy-(9E_11E)-octadecadienoic acid and the PON2 structure did not change much during the simulation time of 20 ns (Fig. 11b), but divaricatacid and 9-hydroxy-(10E)-octadecenoic acid gradually moved away from the PON2 structure.

**Fig. 12:**
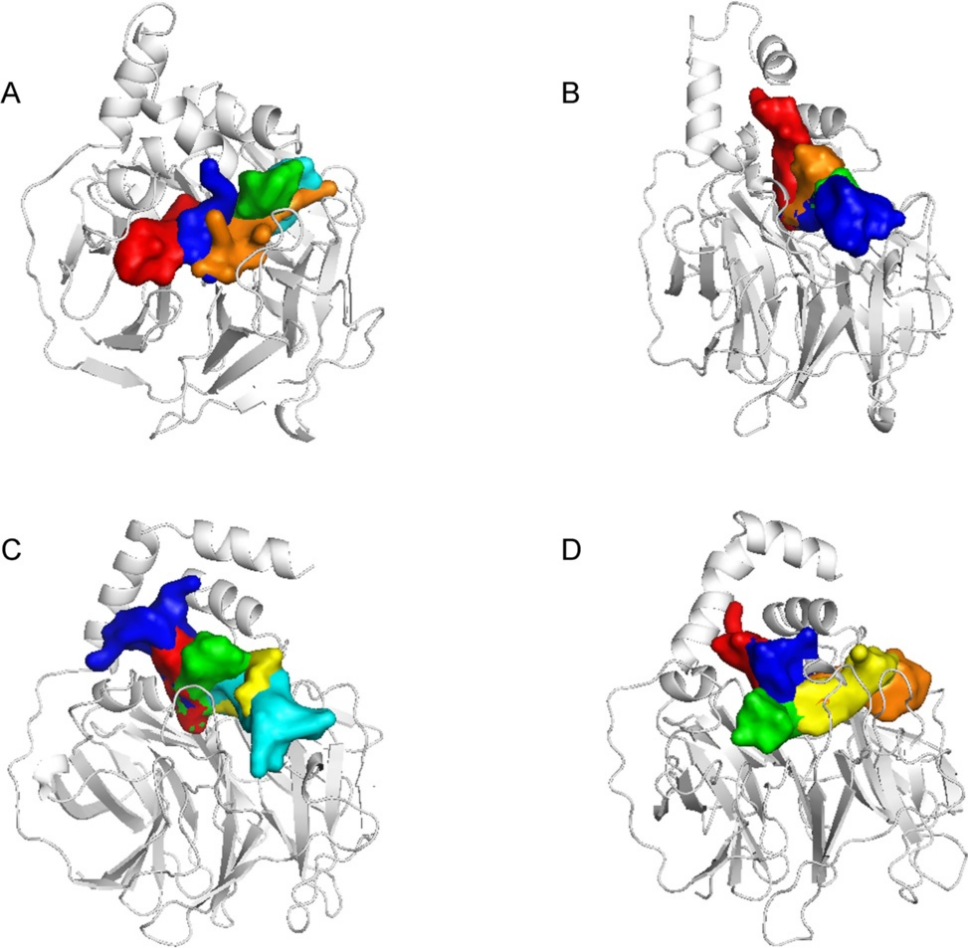
Ligand tunnel prediction for the PON2 system in apo form (**a**) condition and protein-ligand complexes with TCM compounds: (**a**) divaricatacid, (**b**)13-hydroxy-(9E_11E)-octadecadienoic acid, and (**c**) 9-hydroxy-(10E)-octadecenoic acid

**Fig. 13:**
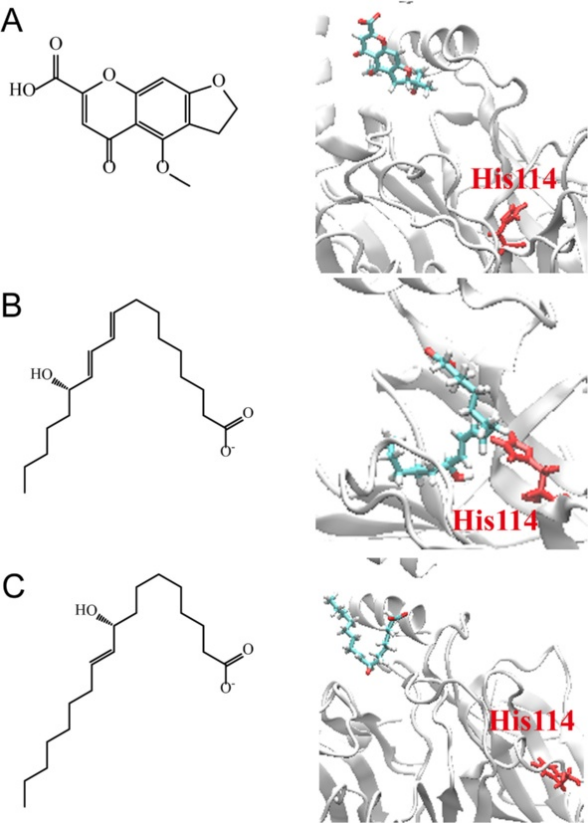
The final snapshots of PON2 with three TCM compounds: (**a**) divaricatacid, (**b**)13-hydroxy-(9E_11E)-octadecadienoic acid, and (**c**) 9-hydroxy-(10E)-octadecenoic acid from the results of MD simulation

We employed CAVER 3.0 software [48] to predict the ligand tunnels in the PON2 structure for the three TCM candidates (Fig. 12). The predicted tunnels are represented by red, blue, green, yellow, cyan and orange solid phases. The apo form of the PON2 structure revealed a broad space of tunnels (Fig. 12a), because there was no docked ligand in the PON2 binding site. A comparison of the MSD values showed that 13-hydroxy-(9E_11E)-octadecadienoic acid was the most stable for all MD simulation times, and hence, the predicted tunnel reveals a narrow space in Fig. 12c, which is represented by the green solid phase. For the other two TCM candidates, divaricatacid and 9-hydroxy-(10E)-octadecenoic acid, the predicted tunnels display spacious areas in Fig. 12b and 12c. In the 20 ns snapshot (Fig. 13), 13-hydroxy-(9E_11E)-octadecadienoic acid is still close to the key residue His114, which confirms the ligand RMSD, migration analysis and the measurement of the protein-ligand distance (Fig. 13b). The final snapshot showed large distances between His114 and both divaricatacid and 9-hydroxy-(10E)-octadecenoic acid (Fig.13a and 13c). This illustrates that 13-hydroxy-(9E_11E)-octadecadienoic acid is the best potential TCM compound to interact with the PON2 structure.

## Discussion

The results of this genome-wide association study identified 22 YZ constitutionally susceptible SNPs, representing 13 regions of 11 chromosomes. Genotypic distribution showed that high YZ scores were significantly associated with *PON2* on chromosome 7, *PIEZO2* on chromosome 18, *ZNF665* on chromosome 19, *FREM2* on chromosome 13, and unknown genes on chromosomes 1p, 2q, and 16p. PCDH10 on chromosome 4q, and unknown genes on chromosome 5q and chromosome 14q were significantly associated with lower risk of the YZ constitution after controlling for age, sex, diabetes duration, and hemoglobin A1c.

Without doubt, the YZ constitution is a consequence of complicated polygenic influences. Genome-wide association studies can provide an overview of whole genomes, and this is an appropriate method for examining the genetic factors of the YZ constitution. This technique had been adapted to explore the genetic base of Korean Sasang constitutional medicine [49].

Common manifestations of YZ include dull, lusterless skin color; dry, cracked, scaly or tough skin; dull purple lips or tongue; and localized pain or numbness. A patient with a YZ constitution tends to express BSS, which according to TCM theory, indicates a morbid state of blood stagnancy in a certain area of the body. The various expressions of BSS are classified according to the severity and area of blood stagnancy. Given its characteristics of circulation disturbance, BSS is considered to be relevant to cardiovascular complications.

The rs7493, rs2299263, and rs17166875 polymorphisms, located in *PON2* on chromosome 7q, belong to one of the paraoxonase (PON) gene families, which encode enzymes participating in the hydrolysis of organophosphates. The PON gene cluster contains 3 adjacent gene members, *PON1, PON2,* and *PON3.* All 3 PON genes share high sequential homology and a similar β propeller protein structure [50] and are thought to have antiatherosclerotic properties. Thus the PON gene cluster has been considered a target in the treatment of atherosclerosis [51, 52]. PON2 has been shown to prevent LDL oxidation, to reverse the oxidation of mildly oxidized LDL, and to inhibit oxidized LDL-induced monocyte chemotaxis [53]. It also increases cholesterol efflux [54] and decreases the size of atherosclerotic lesions [55].

PON2 is a ubiquitously expressed intracellular protein that is expressed in a wide range of tissues [56, 53]. PON2 exhibits antioxidant functions at the cellular level, in addition to a host of intracellular antioxidative enzymes that act against oxidative stress. PON2 is localized in the inner mitochondrial membrane, associated with respiratory complex III, and binds with high affinity to coenzyme Q10. Decreased activity of mitochondrial electron transport chain (ETC.) complexes is implicated in the development of many inflammatory diseases, including atherosclerosis. PON2 protects ETC. complexes against oxidative stress by lowering reactive oxygen species. The intracellular antioxidative effect plays a role in antiatherosclerosis by avoiding endothelial dysfunction caused by mitochondria dysfunction [57, 58]. A common polymorphism rs7493, also known as Ser311Cys, a missense SNP in *PON2*, has also been associated with the risk of CAD [59]. In addition, PON2 plays a role in hepatic insulin signalling. *PON2*-deficient mice display elevated hepatic oxidative stress, coupled with an exacerbated inflammatory response, because of *PON2*-deficient macrophages. *PON2* deficiency is associated with inhibitory insulin-mediated phosphorylation of hepatic insulin receptor substrate-1. PON2 may enhance the influence of the macrophage-mediated inflammatory response in hepatic insulin sensitivity [60]. The *PON2* G148 variant has been associated with elevated fasting plasma glucose in patients with type 2 diabetes [61]. The role of *PON2* provides the genetic basis underlying the YZ constitution. Patients with a strongly YZ constitution may have *PON2* polymorphism with a low protein function which tends to decrease its antioxidative efficacy, resulting in cardiovascular disturbance and hyperglycemia. Thus, *PON2* may be a candidate gene for the YZ constitution. Treatment using herbal medicines or natural compounds that could potentially regulate PON2 might be useful in protecting type 2 diabetes patients with a YZ constitution from cardiovascular complications.

From the docking results of TCM database screening, we chose three potential TCM candidates based on -PMF scores, divaricatacid, 13-hydroxy-(9E_11E)-octadecadienoic acid and 9-hydroxy-(10E)-octadecenoic acid. We further simulated the interaction between PON2 and TCM compounds under dynamic conditions for 20 ns. 13-hydroxy-(9E_11E)-octadecadienoic acid was more stable than the other two candidates for binding with the PON2 structure, which was still connected with active residue His114 after an MD simulation time of 20 ns. According to this result, 13-hydroxy-(9E_11E)-octadecadienoic acid should be a ligand with the ability to regulate PON2. 13-hydroxy-(9E_11E)-octadecadienoic acid is isolated from the seed of *Coix lacryma-jobi L.* Coix oil had been reported the efficacy to decrease adipose tissue and LDL concentrations and increase the total antioxidant capacity in hyperlipidemic rats [62]. 13-hydroxy-(9E_11E)-octadecadienoic acid may play a role in antiatherosclerosis by avoiding endothelial dysfunction by regulating the antioxidant effect of PON2.

The polymorphisms of rs1133146, rs12971799, rs4801958, rs12460170, and rs4803055 are located in the *ZNF665* of chromosome 19q, belonging to the Kruppel zinc finger family. Zinc fingers are the most abundant DNA-binding motifs in humans. The zinc finger protein families are mainly involved in recognizing DNA sequences, but are also able to bind RNA, DNA-RNA hybrids, and even proteins [63]. They work as transcription factors to interact with the control region and achieve gene expression. Kruppel type zinc finger genes are widely present in the human genome, and are usually involved in cell growth and differentiation. To date, specific details of the function of ZNF665 have not been documented. We speculated that the polymorphisms of *ZNF665* might lead to poor gene expression because of poor DNA binding ability, which might disturb cell growth and differentiation; in turn, this disturbance might impede epithelial repair and lead to the dry, cracked, scaly, or tough skin that is characteristic of patients with the YZ constitution. In addition, poor cell growth and differentiation in endothelial progenitor cells might disturb epithelial repair and lead to endothelial dysfunction, which is thought to be a key event in the development of atherosclerosis [64, 65].

The rs12865228 and rs4526895 polymorphisms are located in *FREM2* on chromosome 13q. This gene encodes a membrane protein that belongs to the FRAS1 family. This extracellular matrix protein forms a ternary complex localized on the basement membrane, and plays a role in epidermal-dermal interactions during morphogenetic processes [66]. The protein is thought to be necessary in maintaining the integrity of skin epithelium, vascular stability [67], and the differentiated state of renal epithelia [68]. The polymorphism of *FREM2* in patients with high YZ scores might be implicated in skin changes and microcirculation disturbances, leading to dry, cracked, scaly, tough, and bruised skin, and a dull and lusterless face.

Our study found that SNPs located in *PIEZO2* on chromosome 18p were also associated with high YZ scores among patients with type 2 diabetes. PIZEO2 is a large transmembrane protein with 24 to 36 predicted transmembrane domains, and is a component of the mechanosensitive channel. This channel is required for the rapid adaptation of mechanically activated currents in dorsal root ganglia [69]. Mechanical stimuli drive many physiological processes, including touch and pain sensation, hearing, and blood pressure regulation [70]. Dysfunction of *PIZEO2* might be the source of the abnormal sensations reported by patients with the YZ constitution, including numbness, tightness, tingling pain, and a dull sensation.

*PCDH10* on chromosome 4q was associated with a lower risk of the YZ constitution. *PCDH10* belongs to the protocadherin gene family, a subfamily of the cadherin superfamily. *PCDH10* is a putative tumor suppressor gene [71], and is also known to guide the development of axons [72]. Furthermore, several SNPs located in the intergenic area on chromosomes 1p, 2q, 5q, 14q, and 16p require further investigation to clarify their relationship with the YZ constitution.

## Conclusions

The findings of this study contribute to an understanding of the genetic susceptibility of patients with type 2 diabetes to the YZ constitution. Risk loci occurred in *PON2* that encoded intracellular proteins with antioxidant properties, which normally protect against atherosclerosis and hyperglycemia. Disturbance of this genetic function might constitute one of the mechanisms of cardiovascular disturbance induced by the YZ constitution. Docking and molecular dynamic simulation showed that 13-hydroxy-(9E_11E)-octadecadienoic acid is a stable ligand of PON2 that may have the ability to regulate the antioxidant effects of PON2. Other related genes included *ZNF665*, *FREM2*, *PIZEO2, PCDH10* and several SNPs located in genes of unknown function.

## Additional files

Additional file 1: **Supplementary materials.** Questionnaire items for measuring Yu-Zhi constitution

## Abbreviations

ACR: Albumin-to-creatinine ratio
APM1: Adipose most abundant gene transcript 1
BMI: Body mass index
BSS: Blood stasis syndrome
CAD: Coronary artery disease
CADD: Computer-aided drug design
CI: Confidence interval
DM: Diabetes mellitus
ETC.: Electron transport chain
FRAS1: Fraser syndrome 1
FREM2: FRAS1 related extracellular matrix protein 2
HDL: High-density lipoprotein
LDL: Low-density lipoprotein
MD: Molecular dynamics
MSD: Mean square displacement
OR: Odds ratio
PCDH10: Protocadherin 10
PIEZO2: Piezo-type mechanosensitive ion channel component 2
PLP: Piecewise linear potential
PMF: Potential of mean force
PON: Paraoxonase
PON1: Paraoxonase 1
PON2: Paraoxonase 2
PON3: Paraoxonase 3
PPARD: Peroxisome proliferator-activated receptors delta
PPARG: Peroxisome proliferator-activated receptors gamma
RMSD: Root mean square deviation
RMSF: Root mean square fluctuation
SNP: Single nucleotide polymorphism
TCM: Traditional Chinese medicine
UTR: Untranslated region
VDW: Van der Waals
YZ: Yu-Zhi
ZNF665: Zinc finger protein 665
