# Supplementary material for: Susceptible gene of stasis-stagnation constitution from genome-wide association study related to cardiovascular disturbance and possible regulated traditional Chinese medicine
Source: BMC Complement Altern Med. 2015 Jul 14;15:229. doi: 10.1186/s12906-015-0761-x (PMC4501203; doi:10.1186/s12906-015-0761-x)
Supplement: Supplementary file 1 — Supplementary materials. Questionnaire items for measuring Yu-Zhi constitution [file 12906_2015_761_MOESM1_ESM.doc]

Supplementary materials

| Questionnaire items for measuring Yu-Zhi constitution |
| --- |
| 1. I feel numbness in the limbs. |
| 1. I feel chest tightness or my chest seems to be oppressed by something. |
| 1. There is tingling pain in my body which makes me uncomfortable. |
| 1. I have a dull sensation or pain over the lateral side (costal region) of my body. |
| 1. Bruises appear on my skin without an apparent cause. |
| 1. My skin gets dry, cracked, scaly, or tough. |
| 1. My face feels dull and lustreless. |
| 1. My lips or tongue have a dull purple color, or I find petechiae on them. |
